# Design Methodology of a New Wavelet Basis Function for Fetal Phonocardiographic Signals

**DOI:** 10.1155/2013/505840

**Published:** 2013-05-23

**Authors:** Vijay S. Chourasia, Anil Kumar Tiwari

**Affiliations:** ^1^Manoharbhai Patel Institute of Engineering & Technology, Kudwa, Gondia, Maharashtra 441 614, India; ^2^Indian Institute of Technology, Rajasthan MBM College Campus, Old Residency Road, Ratanada, Jodhpur, Rajasthan 342 011, India

## Abstract

Fetal phonocardiography (fPCG) based antenatal care system is economical and has a potential to use for long-term monitoring due to noninvasive nature of the system. The main limitation of this technique is that noise gets superimposed on the useful signal during its acquisition and transmission. Conventional filtering may result into loss of valuable diagnostic information from these signals. This calls for a robust, versatile, and adaptable denoising method applicable in different operative circumstances. In this work, a novel algorithm based on wavelet transform has been developed for denoising of fPCG signals. Successful implementation of wavelet theory in denoising is heavily dependent on selection of suitable wavelet basis function. This work introduces a new mother wavelet basis function for denoising of fPCG signals. The performance of newly developed wavelet is found to be better when compared with the existing wavelets. For this purpose, a two-channel filter bank, based on characteristics of fPCG signal, is designed. The resultant denoised fPCG signals retain the important diagnostic information contained in the original fPCG signal.

## 1. Introduction

Continuous and long-term fetal monitoring has become an essential approach for better accuracy in diagnosis [1]. Variations in fetal heart rate (FHR) provide up-to-date information about the fetal health status [2]. Doppler ultrasound based fetal cardiotocography (fCTG) is currently being used as a technique for recording and analysis of the FHR. In this technique, a graph of fetal heart rate (cardio-) and uterine contractions (-toco-) are recorded during pregnancy [3]. This technique cannot be used for long-term monitoring of the fetus due to several reasons [4] such as the cost and complexity of the monitoring instrument, ultrasound radiation exposure, and gel application.

Fetal phonocardiography, recording of vibroacoustic (fPCG) signals from the maternal abdominal surface, may become an important alternative to fCTG [5]. However, these fPCG signals are heavily contaminated by noise from various sources [6–11]. Hence to extract important diagnostic information such as FHR, frequency/pitch, intensity, timing, and energy of fetal heart sound, this technique requires matured signal processing strategies. 

Researchers from biomedical signal processing community have applied several techniques for denoising of fPCG signals. Varady (2001) presented a wavelet-based denoising method for phonocardiographic signals using two-channel signal recording and an adaptive cross-channel coefficient thresholding technique [12]. Messer et al. (2001) attempted to answer about which wavelet families, levels of decomposition, and thresholding techniques best removes the noise in a PCG [13]. Jianfeng et al. [5] used a modified spectral subtraction algorithm to remove the unwanted stationary background noise from the noisy fetal heart sound. The AM/FM modulation technique is employed to make the fetal heart sound more audible so that both pregnant women and gynecologists can identify the rhythmic fetal heart beat sounds easily [5]. Mittra et al. [14] compared and analyzed the performance of various digital noise cancellation techniques for fetal heart sound. Their experimental results show that adaptive filtering using Recursive Least Squares (RLS) algorithm is an appropriate methodology for the fetal phonocardiographic signals denoising implementation [14]. Tosanguan et al. (2008) proposed a 2-channel signal processing technique, termed interference suppression via spectral comparison (ISSC) and aims at improving the quality of the recorded heart sound or the PCG data [15]. Zhao et al. (2009) developed a denoising method for heart sound signal using improved thresholding function in wavelet domain [16]. Chourasia et al. (2010) presented a technique for denoising of fPCG signals using wavelet transform [17]. In another work, Chourasia et al. (2012) also developed a methodology for removal of unwanted noise from the fPCG signal using nonnegative matrix factorization [18].

The above-mentioned researchers have used conventional filtering techniques and wavelet transform for denoising the fPCG signals. The conventional techniques also impact on the useful signals and hence may result in loss of information of diagnostic importance. Additionally, the passed band may still contain noise. In view of these limitations of conventional methods of denoising, wavelet threshold denoising has been applied to denoise heart sound recordings. The wavelet based denoising method try to preserve the signal by operating only on those selected regions of the bandwidth that need filtering. This technique requires appropriate selection of wavelet family, level of decomposition, and the method to be used for calculation of threshold. The existing works are limited only to employ the existing wavelet family from the bank of wavelet transform. 

This present work proposes a novel algorithm for denoising of fPCG signals using wavelet transform (WT). In this approach, a new wavelet family and its mother wavelet are developed. For this purpose, a quadratic mirror filter (QMF) bank [19] is designed based on the characteristics of the fPCG signals. This filter bank requires a low-pass and a high-pass filter in decomposition (analysis) phase and reconstruction (synthesis) phase. Appropriate denoising algorithm and thresholding rule have been selected and used with the developed mother wavelet. The developed wavelet family provides better results when compared with the existing wavelets. The obtained denoised fPCG signals retain the vital diagnostics information contained in the original signals.

The rest of the paper is organized as follows. A brief introduction about fetal phonocardiographic signals and wavelet theory has been provided in Sections 2 and 3, respectively.  Section 4 discusses the selection of appropriate denoising algorithm and thresholding rule for fPCG signals. Design methodology of filter bank for new wavelet has been explained in Section 5. Finally, the experimental results are described in Section 6, followed by conclusion and discussion in Section 7.

## 2. Fetal Phonocardiographic Signals

Fetal phonocardiography is the recording of natural vibroacoustic signals from the maternal abdominal surface. The fPCG signal carries valuable information concerning the physiological state of unborn [20]. It is also capable of recognizing additional potential dysfunctional signals of the fetal heart such as those related to cardiac murmurs, split effect, and breathing movements, which can be detected from the analysis of abdominal sound (fPCG) signals. However these cardiac anomalies are impossible to detect with the widely accepted fCTG technique due to its principle of operation [21]. Additionally, phonocardiography provides a permanent and lasting record of fetal heart sounds which by comparison at a later stage may prove to be of great prognostic importance. The fPCG technique has been introduced earlier but overlooked by biomedical scientists and medical experts mainly due to its low signal-to-noise ratio (SNR) [22]. The fPCG signals are a linear summation of [23]

- fetal heart sound,
- internal noise,
- external noise.

**Figure 1:**
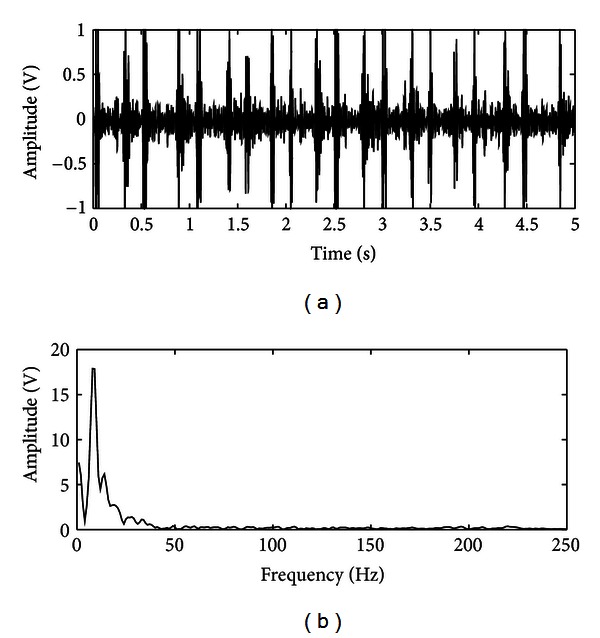
(a) Typical fPCG signal and (b) its frequency spectrum.

The fetal heart sound is a signal produced by mechanical activity of the fetal heart. The fetal heart is basically divided into two pairs of chambers and has four valves: the mitral and tricuspid valves. In the fetal cardiac cycle, when the ventricles begin to contract, the blood attempts to flow back into the lower pressure atrial chambers. This reverse flow of blood is arrested by the shutting of the mitral and tricuspid valves, which produces the first heart sound (S1). Whenever the pressure in the ventricular chambers becomes too high for the pulmonary valves to withstand, they open, and the pressurized blood is rapidly ejected into the arteries. While the ventricles are being evacuated, the pressure of the remaining blood decreases with respect to that in the arteries. This pressure gradient causes the arterial blood to flow back into the ventricles. The pulmonary valves arrest this reverse flow by shutting, which gives rise to the second heart sound (S2) [24]. The frequency spectrum of fetal heart sound lies below 200 Hz. Figures 1(a) and 1(b) show a typical fetal phonocardiographic (fPCG) signal and its frequency spectrum, respectively.

The internal noise is a random signal caused due to maternal respiratory sounds, acoustic noise produced by the fetal movement, maternal digestive sound, maternal respiratory sound, maternal heart sound, and placental blood turbulence. These noises are of low amplitude with main frequency components from 0 to 25 Hz [4].

Similarly, the external noise is a combination of shear noise from movement of the sensor during recording and ambient noise originating from the environment such as sound produced by fan, air conditioner, and hue and cry of the nearby people. It is comparatively of high amplitude and frequency (100–20000 Hz) [4].

## 3. Theoretical Background

### 3.1. Wavelet Transform

The WT is a two-dimensional time-scale processing method for nonstationary signals with adequate scale values and shifting in time. The major advantage of the WT is that it has a varying window size, being broad at low frequencies and narrow at high frequencies, thus leading to best possible time-frequency resolution in all frequency ranges [25, 26]. The analysis of nonstationary signals requires proper location of transitions or discontinuities and identification of their long-term behavior. The WT represents a time function in terms of simple and fixed building blocks derived from mother wavelet by translation and dilation operations. The translation is the shifting of mother wavelet along the time axis while dilation or scaling starches or compresses it.

The WT can be categorized as continuous wavelet transform (CWT) or discrete wavelet transform (DWT). The CWT is defined as the convolution between the original signal *s*(*t*) and a wavelet *ψ*(*t*) which can be calculated by

(1) CWTψ(a,b)=∫−∞+∞s(t)ψa,b∗(t)dt=1a  ∫−∞+∞s(t)ψ∗(t−ba)dt,

where CWT_*ψ*_(*a*, *b*) is a continuous wavelet transform, “*s*(*t*)” is a signal under study, “*a*” is a scale coefficient connected with stretching or compression of signal in time, “*b*” is a shift connected with time location, “*ψ*_*a*,b_*(*t*)” is a wavelet function or mother wavelet representing a wavelet family, “*” denotes the complex conjugation, and the factor $1/\sqrt{a}$ is used for energy normalization purposes so that the transformed signal will have the same energy at every scale. In CWT, the scaling parameter *a* and translation parameter *b* change with time continuously. Hence wavelet coefficients are calculated for every possible scale, which requires huge processing power and results in large amount of data. 

The Discrete Wavelet Transform (DWT) coefficients are usually sampled from the CWT on a dyadic grid, choosing parameters of translation *b* = k*2^−*j*^ and scale *a* = 2^−*j*^. Where *j*, *k* ∈ *Z* a set of positive integers and *k* = 0,1,…, *n* − 1, *n* represents the number of samples. These dilation and translation parameters are discretized leading to the DWT. After discretization, the wavelet function is defined as

(2) $$\begin{array}{c} {\text{DWT}}_{\psi }\left(j,k\right)=\int_{-\infty }^{+\infty }s\left(t\right)\psi_{j,k}^{*}\left(t\right)dt. \end{array}$$

Here *ψ*_*j*,k_*(*t*) is the dilated and translated version of the wavelet function and given as

(3) $$\begin{array}{c} \psi_{j,k}\left(t\right)=2^{j/2}\psi \left(2^{j}t-k\right), \end{array}$$

where *ψ* is called as mother wavelet and *ψ*_*j*,*k*_ is called as daughter wavelet. The level *j* determines how many wavelets are needed to cover the mother wavelet, and the number *k* determines the position of the wavelet and gives the indication of time. DWT analyzes the signal by decomposing it into its coarse and detail information, which is accomplished by using successive high-pass and low-pass filtering operations, on the basis of the following equations:

(4) yhigh(k)=∑ns(n)·h(2k−n),ylow(k)=∑ns(n)·g(2k−n),

where *y*_high_(*k*)  and *y*_low_(*k*) are the outputs of the high-pass and low-pass filters with impulse response *h* and *g*, respectively, after downsampling by 2 [27, 28]. The coefficients of low-pass filter are called “approximation” (*c*_*j*,*k*_) and coefficients from high-pass filter are called “detail” (*b*_*j*,*k*_) wavelet coefficients. The detail coefficients are defined by the following equation:

(5) $$\begin{array}{c} b_{j,k}=\int s\left(t\right)\psi_{j,k}^{*}\left(t\right)dt, \end{array}$$

where *ψ*_*j*,*k*_ are wavelet functions given by

(6) $$\begin{array}{c} \psi_{j,k}\left(t\right)=\frac{1}{\sqrt{2^{j}}}\;\psi \left(\frac{t-k2^{j}}{2^{j}}\right). \end{array}$$

Similarly, the approximate coefficients are

(7) $$\begin{array}{c} c_{j,k}=\int s\left(t\right)\Phi_{j,k}^{*}\left(t\right)dt, \end{array}$$

where Φ_*j*,*k*_ are called scaling functions as follows:

(8) $$\begin{array}{c} \Phi_{j,k}\left(t\right)=\frac{1}{\sqrt{2^{j}}}\;\Phi \left(\frac{t-k2^{j}}{2^{j}}\right). \end{array}$$

The discrete inverse transform is found by adding the translated, dilated wavelets, weighted by the coefficients:

(9) $$\begin{array}{c} f\left(t\right)=\sum_{j,k}b_{j,k}\psi_{j,k}\left(t\right). \end{array}$$

The DWT gives a multiresolution description of a signal which is very useful in analyzing real-time signals [29].

The general wavelet denoising procedure is described in the following steps:

- decomposition of the fPCG signal using DWT to obtain the approximation and detail coefficients,
- thresholding of these decomposed coefficients using an appropriate denoising algorithm,
- reconstruction of the fPCG signal from these thresholded coefficients using the inverse transform (IDWT) [17].

### 3.2. Denoising Algorithm

The *denoising algorithm* uses statistical regression of noisy coefficients over time to obtain a nonparametric estimation of the reconstructed signal without noise. The thresholding algorithms commonly employed for denoising of the nonstationary signals are [30]

- universal threshold,
- minimax threshold,
- rigorous Stein's Unbiased Risk Estimate (SURE) threshold.

*(i) Universal Threshold (Sqtwolog).* The universal threshold denoising algorithm is a fixed threshold method which can be calculated by

(10) $$\begin{array}{c} \lambda =\sigma \sqrt{2\log\left(n\right)}, \end{array}$$

where *n* denotes the length of the signal and *σ* is the standard deviation.

*(ii) Minimax Threshold (Minimaxi).* Minimax threshold also uses fixed threshold and it yields minimax performance for Mean Square Error (MSE) against an ideal procedures. This threshold level depends on the noise and signal relationships in the input data and it is given by *λ* = *σλ*_*n*_, where *λ*_*n*_ is determined by a minimax rule such that the maximum risk of estimation error across all locations of the data is minimized.

*(iii) Rigorous SURE Threshold (Rigrsure).* The denoising algorithms described previously use global thresholds. That is, the computed threshold is applied to all wavelet coefficients. The rigorous SURE threshold algorithm describes a scheme that uses a threshold value *λ*_*j*_ at each resolution level *j* of the wavelet coefficients. This algorithm is also known as SureShrink and uses the Stein's Unbiased Risk Estimate (SURE) criterion to get an unbiased estimate.

These denoising algorithms can be divided into linear and nonlinear methods. The linear method is independent of the size of empirical wavelet coefficients, and therefore the size of the coefficient by itself is not taken into account. It assumes that signal noise can be found mainly in fine scale coefficients and not in coarse scales. The nonlinear method is based on the idea that the signal noise can be found in every coefficient and is distributed over all scales.

### 3.3. Wavelet Thresholding Rules

*Wavelet thresholding* is a signal estimation technique that exploits the capabilities of wavelet transform for signal denoising [31]. This method has been researched extensively due to its effectiveness and simplicity. Any signal *s*(*t*) can be represented by the summation of the original signal *x*(*t*) and the noise *n*(*t*) as follows:

(11) $$\begin{array}{c} s\left(t\right)=x\left(t\right)+n\left(t\right). \end{array}$$

**Figure 2:**
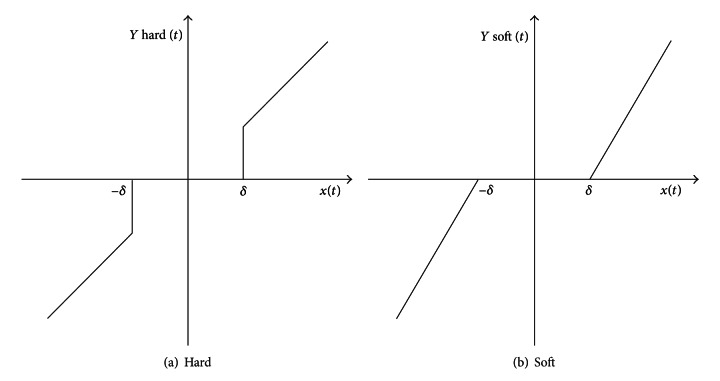
Wavelet thresholding.

After performing the wavelet transform

(12) $$\begin{array}{c} S_{j,k}=X_{j,k}+N_{j,k}, \end{array}$$

where *S*_*j*,*k*_ is the *k*th wavelet coefficient in the scale *j*. There are two ways of thresholding with threshold *λ*; the shapes of these thresholding operators are illustrated in Figure 2.

*Hard Thresholding *(*h*). In hard thresholding, those wavelet coefficients with absolute values below or at the threshold level (*λ*) are affected only and they are replaced by zero value whereas others are kept unchanged

(13) $$\begin{array}{c} {\hat{Y}}_{j,k}^{\text{hard}}=\{\begin{array}{cc} Y_{j,k} & \text{for}\;\;\left|Y_{j,k}\right|>\lambda \\ 0 & \text{for}\;\;\left|Y_{j,k}\right|\leq \lambda . \end{array} \end{array}$$

*Soft Thresholding (s).* In soft thresholding, coefficients above threshold level (*λ*) are also modified; they are reduced by particular value of the threshold

(14) $$\begin{array}{c} {\hat{Y}}_{j,k}^{\text{soft}}=\{\begin{array}{cc} Y_{j,k}-\lambda & \text{for}\;\;Y_{j,k}\geq \lambda \\ 0 & \text{for}\;\;\left|Y_{j,k}\right|<\lambda \\ Y_{j,k}+\lambda & \text{for}\;\;Y_{j,k}\leq -\lambda . \end{array} \end{array}$$

Hard thresholding maintains the scale of the signal but introduces ringing and artifacts after reconstruction due to a discontinuity in the wavelet coefficients. Soft thresholding eliminates this discontinuity resulting in smoother signals but slightly decreases the magnitude of the reconstructed signal.

## 4. Selection of Denoising Algorithm and Thresholding Rule for fPCG Signals

The presented work also contributes in the selection of suitable algorithm and thresholding rule for denoising of fPCG signals. Based on the discussion in Sections 3.2 and 3.3, there are three denoising algorithms, namely, universal threshold, minimax threshold, and rigorous SURE threshold and two thresholding rules which can be employed in denoising of fPCG signals. These denoising algorithms and thresholding rules are practically implemented and their results are compared. The mean squared error (MSE) is used to evaluate the performance of all denoising algorithms and thresholding rules in denoising the fPCG signals. It can be obtained using following expression:

(15) $$\begin{array}{c} \text{MSE}=\frac{\sum_{i=1}^{n}{\left(s-s_{e}\right)}_{i}^{2}}{n}, \end{array}$$

where *n* denotes the length of the signal, *s* represents the original signal and *s*_*e*_ is the estimated signal obtained from the denoised wavelet coefficients.

**Figure 3:**
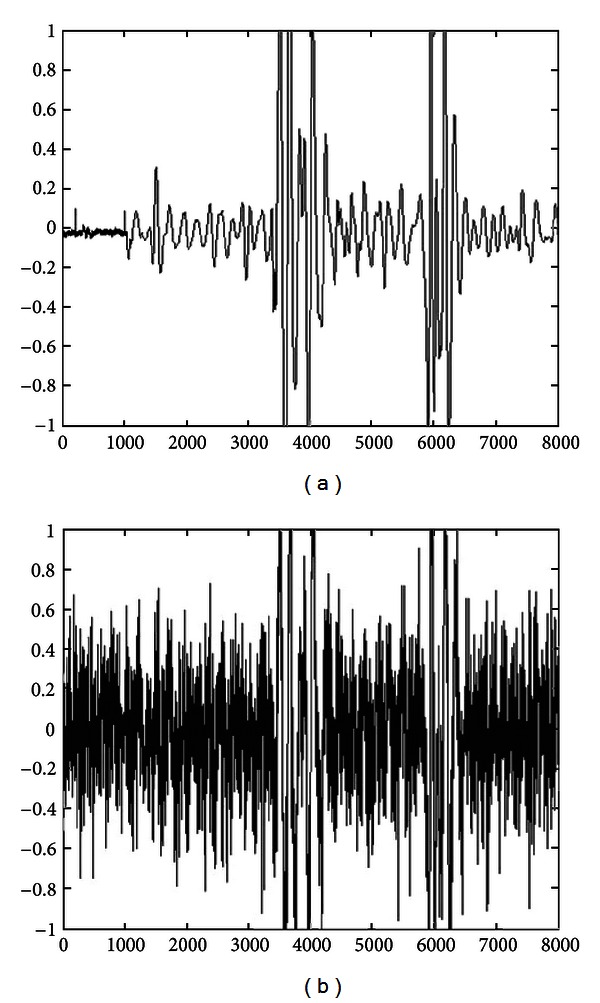
Waveform of (a) reference fPCG signal and (b) test fPCG signal with additive noise.

Figure 3(a) shows the waveform of one simulated fPCG signal as an example. This signal is used as a reference signal in the selection process of appropriate denoising algorithms and wavelet threshold.  Figure 3(b) is a test signal generated by adding simulated stationary random noise in the original fPCG signal which is shown in Figure 3(a). The noise is generated by recording it, first, in actual conditions. After recording, the noise signal is simulated with similar average characteristics. The simulated noise is then mixed with the fPCG signal shown in Figure 3(a) and used as an input to evaluate the performance of the selection of appropriate denoising algorithm and thresholding rule.

**Figure 4:**
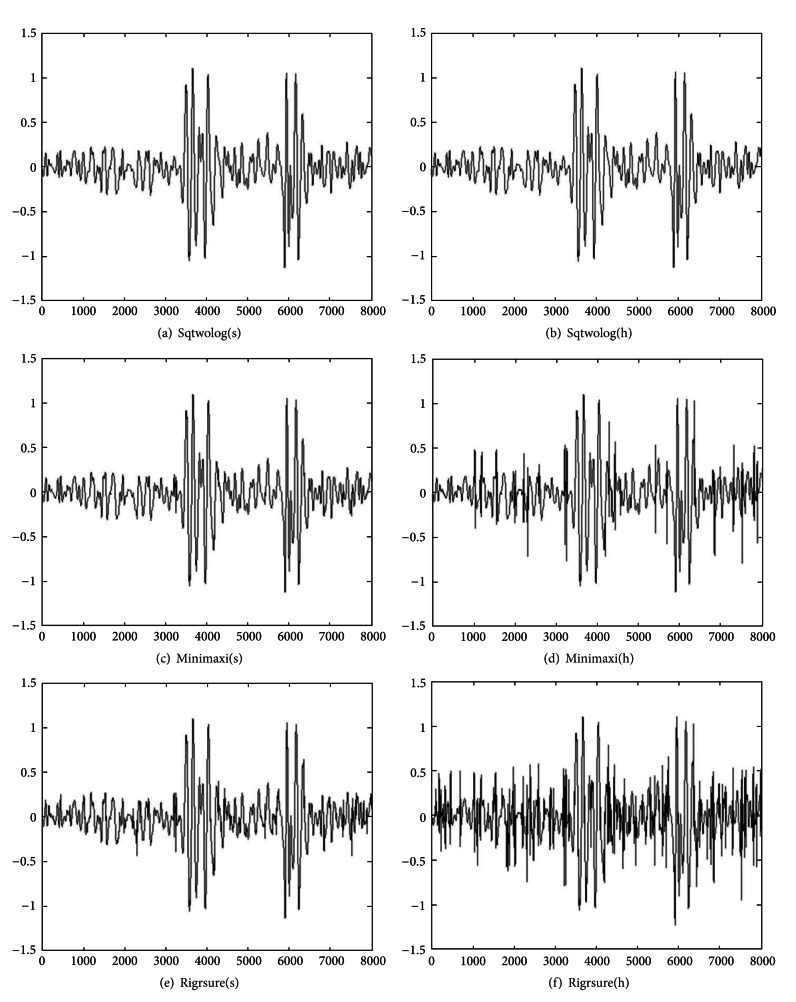
Denoised fPCG signal using different algorithms: (a) sqtwolog(s), (b) sqtwolog(h), (c) minimaxi(s), (d) minimaxi(h), (e) rigrsure(s), and (f) rigrsure(h).

The test signal so obtained is analyzed with DWT based multiresolution analysis. The signal is decomposed to five levels using fourth order Coiflets wavelet. This selection of mother wavelet is based on the fact that it possesses all the properties needed for analysis of the fPCG signals [29]. All the three algorithms with soft or hard thresholding rule are applied for denoising of the fPCG signal. The resultant waveforms from this implementation are shown in Figure 4, and the best estimations obtained are depicted in Table 1. In Figures 3 and 4, the *x*-axis represents number of samples and the *y*-axis represents relative amplitude.

Table 1 shows a comparison of three denoising algorithms with soft or hard thresholding rule. The rigorous SURE threshold algorithm with soft thresholding rule yields the best estimation with considerably smaller MSE as compared to the other algorithms for denoising of fPCG signals.

## 5. Design of Filter Bank for New Wavelet

Since it has been discussed earlier in Section 1 that the effective implementations of wavelet transform in denoising the fPCG signals requires appropriate wavelet basis function, we propose to design a new wavelet function which will be based on the characteristics of the fPCG signals and hence will improve the performance of its denoising. The main properties to be verified in designing a family of wavelet are existence of scaling function, symmetricity and compactly support of wavelet and scaling functions, availability of filter bank, orthogonality of filter bank, and smoothness of wavelet function. With these properties, the wavelet family may be called as suitable wavelet for the analysis of intended signal. This wavelet will lead to maximization of wavelet coefficient values to produce the highest local maxima of the signal in wavelet domain. It also produces the possibility of best characterization of frequency content of that signal [32].

In view of these considerations, we developed a new wavelet function which is orthogonal and named as “fetal.” In orthogonal wavelet analysis, the number of convolutions at each scale is proportional to the width of the wavelet basis at that scale. This produces a wavelet spectrum that contains discrete “blocks” of wavelet power and is useful for signal processing as it gives the most compact representation of the signal [33]. Conversely, a nonorthogonal analysis is highly redundant at large scales, where the wavelet spectrum at adjacent times is highly correlated. The new wavelet also has a small number of coefficients in high-pass subbands and allows the signal singularities, transitions, and edges intact in the low-pass subband. 

**Figure 5:**
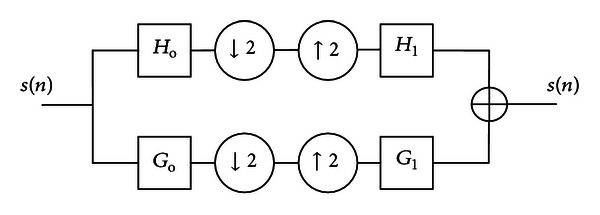
Two-channel filter bank.

To synthesize new wavelet it requires a two-channel filter bank which has a low-pass and a high-pass filter in decomposition (analysis) phase and reconstruction (synthesis) phase. This two-channel multirate filter bank consists of filters which process the input signal at half of its original rate. A block diagram of two-channel filter bank is shown in Figure 5. The signal *s*(*n*) gets filtered by *G*_o_ and *H*_o_ filters and downsampled. These filters are called analysis or decomposition filters. The output of these filters contains the signal at half rate. These are called subbands of the signal. Each subband can be further divided into smaller subbands using the same filter bank. After being processed, the signal is upsampled and filtered using *G*_1_ and *H*_1_ filters, which are called synthesis or reconstruction filters. For perfect reconstruction, in a two-channel filter bank, the downsampled signal that contains only the even samples is given to the first channel and the downsampled signal that contains only the odd samples is given to the second channel. This separation of signal into even and odd components is called polyphase representation of the signal.

Four important characteristics are taken into account while designing the filter bank: (i) perfect reconstruction, (ii) orthogonality of the filter bank and the underlying wavelet based multiresolution structure, (iii) flatness of the filters and vanishing moments in the wavelets, and (iv) smoothness of the wavelets. The output of a perfect reconstruction two-channel filter bank is [34]

(16) Y(z)=12[Go(z)G1(z)+Ho(z)H1(z)]X(z)+12[Go(−z)G1(z)+Ho(−z)H1(z)]X(−z),

where *X*(*z*), *G*(*z*), and *H*(*z*) are the *z*-transform of the input signal, analysis filters and synthesis filters respectively. For perfect reconstruction: firstly, the alias term *X*(−*z*) must be zero, hence

(17) $$\begin{array}{c} G_{\text{o}}{\left(-z\right)G}_{1}\left(z\right)+H_{\text{o}}{\left(-z\right)H}_{1}\left(z\right)=0. \end{array}$$

This can be achieved by letting

(18) $$\begin{array}{c} G_{1}\left(z\right)=H_{\text{o}}\left(-z\right),\;\;H_{1}\left(z\right)={-G}_{\text{o}}\left(-z\right). \end{array}$$

Second, the distortion term must be constant or a pure delay time; that is,

(19) $$\begin{array}{c} G_{\text{o}}{\left(z\right)G}_{1}\left(z\right)+H_{\text{o}}{\left(z\right)H}_{1}\left(z\right)=2z^{-l}, \end{array}$$

where *l *denotes a time delay.

Equation (18) can also be written as

(20) $$\begin{array}{c} H_{\text{o}}\left(z\right)=G_{1}\left(-z\right),\;\;H_{1}\left(z\right)={-G}_{\text{o}}\left(-z\right). \end{array}$$

Substituting this in (19)

(21) $$\begin{array}{c} G_{\text{o}}{\left(z\right)G}_{1}\left(z\right)-G_{1}{\left(-z\right)G}_{\text{o}}\left(-z\right)=2z^{-l}, \end{array}$$

(22) $$\begin{array}{c} P_{\text{o}}\left(z\right)-P_{\text{o}}\left(-z\right)=2z^{-l}, \end{array}$$

where *P*_o_(*z*) denotes the product of two low-pass filters *G*_o_(*z*) and *G*_1_(*z*). Equation (21) indicates that all odd terms of product of two low-pass filters must be zero for order *l*, where *l* must be odd and even order terms are arbitrary [35]. Hence it can be written as

(23) $$\begin{array}{c} P_{\text{o}}\left(n\right)=\{\begin{array}{cc} 0 & n\;\text{odd},\;\;n\neq l\\ 2 & n=l\\ \text{arbitrary} & n\;\text{even}. \end{array} \end{array}$$

Hence it can be concluded that the design process of two-channel filter bank for a new wavelet is reduced into two steps as follows:

1. design of filter *P*_o_(*z*) which satisfies (23),
2. factorize *P*_o_(*z*) into *G*_o_(*z*) and *G*_1_(*z*).

**Figure 6:**
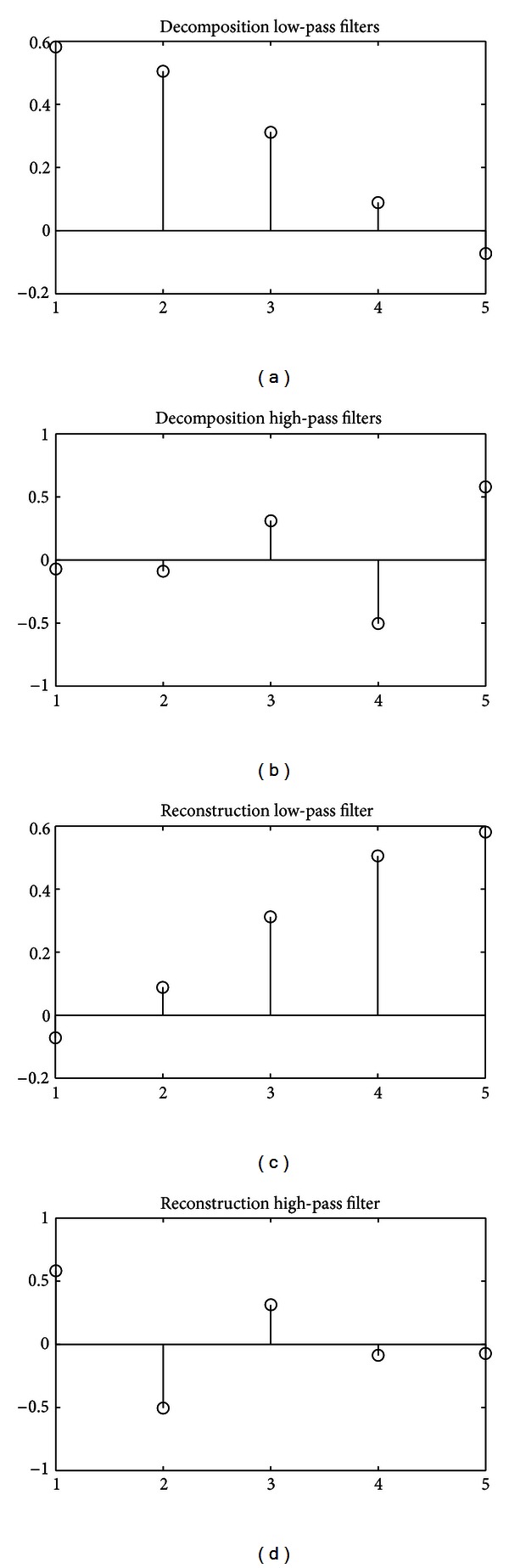
Impulse response for the reconstruction and decomposition filters of fetal.

In this work, the filter *P*_o_(*z*) is designed based on the characteristics of fPCG signals. The common requirements of this design are linear phase, minimum phase, and orthogonality of the filter. A fourth-order low-pass Butterworth filter is chosen because the transition width requirement is not stringent for the given cut-off frequency. This will also help in reducing the computational complexity. The Butterworth filter satisfies the conditions for perfect reconstruction. It has linear frequency response in the pass band as compared to Chebyshev Type I/Type II and elliptic filters. The filter *P*_o_(*z*) is factorized into *G*_o_(*z*) and *G*_1_(*z*), and then the coefficients of *H*_o_(*z*) and *H*_1_(*z*) are derived using (20) [32, 36].  Figure 6 shows the impulse response of the four filters computed for construction of filter bank of the new wavelet “fetal.”

**Figure 7:**
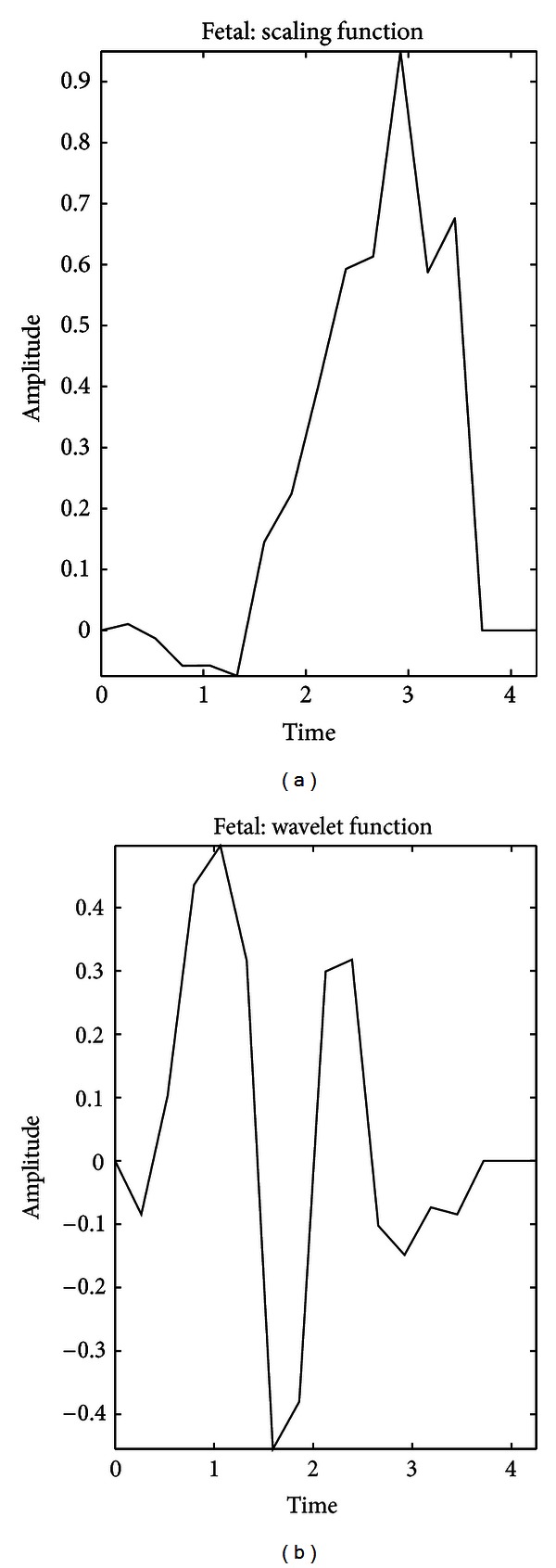
Wavelet and scaling function of “fetal” wavelet.

The wavelet and scaling functions are then derived from the coefficients of these filters using (6) and (8), respectively.  Figure 7 shows the wavelet and scaling functions of the “fetal” wavelet.

With these wavelet and scaling functions, the wavelet and scaling coefficients for multiresolution analysis are obtained. The developed wavelet “fetal” is now ready to use. All the discrete analysis functions, including dwt, idwt, and wavedec. can operate on the new wavelet. Similarly, all the continuous analysis functions, including cwt, wscalogram, and the corresponding GUI tools, can also operate on the new wavelet. In this work, this new wavelet “fetal” is used for intended denoising of the fPCG signals.

## 6. Experimental Results

**Figure 8:**
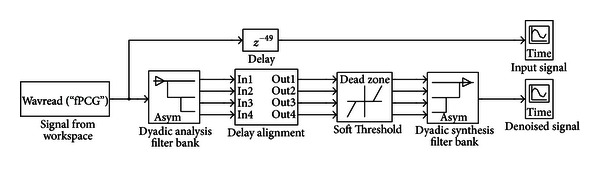
Simulink model for wavelet denoising.

The newly developed wavelet “fetal” is used for denoising of real-time fetal heart sound signals which are of widely distinct nature and quality. A Simulink model is developed for computer based denoising of these signals [37].  Figure 8 shows the model for wavelet denoising of the fPCG signal through which the filters of developed wavelet family and selected threshold criterion are implemented.

In this model, the fPCG signals are fetched from the work space and applied to the Dyadic analysis filter bank. These signals carry fetal heart sound and a damped version of simulated maternal organs' sounds along with the external noise. The analysis filter bank decomposes the fPCG signals into a collection of subbands with smaller bandwidths and slower sample rates. This bank uses a series of high-pass and low-pass FIR filters to repeatedly divide the input frequency range. The fPCG signals are decomposed to the 3 levels by newly developed “fetal” wavelet with the analysis filter bank. These decomposed wavelet coefficients consist of details in the input fPCG signals. The denoising of fPCG signals is carried out by using selected algorithm (Rigorous SURE) and thresholding rule (Soft threshold). The synthesis filter bank reconstructs the signal decomposed by the analysis filter bank block. This bank takes in subbands of this signal and uses them to reconstruct the signal by using a series of high-pass and low-pass FIR filters. The reconstructed signals have a wider bandwidth and faster sample rate than the input subbands. The waveforms of input signal and denoised outputs are displayed through “Time Scope” blocks.

For performance evaluation of the developed wavelet, five test fPCG signals (S1–S5) are generated using a signal simulation module (SSM) [38]. These test signals are denoised using some existing competitive wavelets families and the developed wavelet. The competitive wavelets are the one that possess required properties of orthogonality and a scaling function Φ such as Daubechies, Symlets, and Coiflets. The results of this comparison are computed using MSE equation (15) and presented in Table 2. As explained earlier, all the three denoising algorithms with soft or hard threshold are used for denoising of the fPCG signals.

The results in Table 2 show that the developed wavelet “fetal” along with rigorous SURE algorithm and soft threshold provides the best performance for denoising the fPCG signals.

The developed wavelet family was also implemented for denoising of a real fPCG signal. This signal was recorded using a specially developed wireless data acquisition system [39]. The recording of fPCG signal was obtained in a quiet room with the help of a medical expert and a trained nurse. A pregnant woman visiting for antenatal care was requested to contribute in the real-time testing of the developed system. The subject was having 34 weeks of gestation and singleton pregnancy. She was asked to lie in the supine position, with the head resting on a pillow. The phonocardiographic sensor was positioned on the abdominal surface of the mother and adjusted to acquire the maximum intensity of the signal. An abdominal belt was used to fasten the sensor. The signal was sampled with sampling frequency of 8000 Hz, 16 bit resolution and saved for further processing.

**Figure 9:**
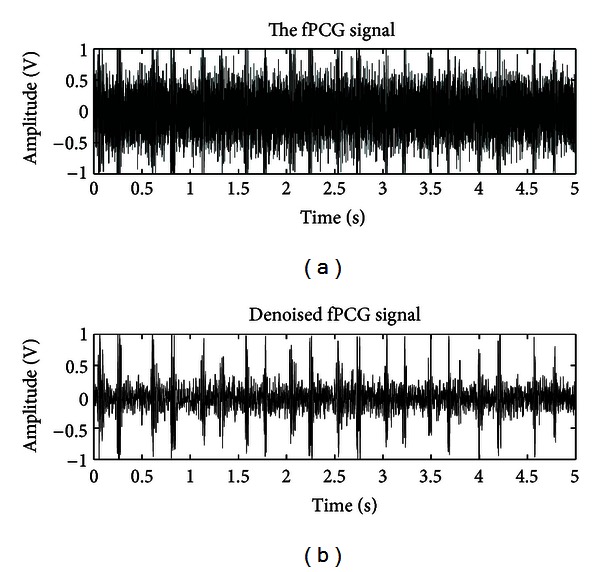
Waveforms of a real fPCG signal and its denoised version.

The recorded fPCG signal is now fetched from the memory through the model, as shown in Figure 8, for denoising.  Figure 9 shows the waveforms of original and denoised version of this signal. In these waveforms, *x*-axis represents the time in seconds, whereas *y*-axis represents amplitude of signal in volts.

## 7. Discussion and Conclusion

The fPCG signals are of very low amplitude and contain poor signal-to-noise ratio. The main sources of noise are maternal biological activities, external noises such as sound produced by electrical appliances, movement of transducer, and so forth. These noises show overlapping spectra with the actual fPCG signals. Hence conventional noise removal techniques are not suitable for denoising of these signals. During denoising, care has to be practiced to preserve the features contained in the original signal. These preserved features are relevant and necessary for an appropriate diagnosis about fetal health.

In this paper design of a new wavelet basis function for denoising of fPCG signals has been carried out. The key features of newly designed family are its speed of convergence at infinity to 0, regularity, and orthogonality. It also has a small number of coefficients in high-pass subbands and allows the signal singularities, transitions, and edges intact in the low-pass subband. It has been found that the combination of optimal perfect reconstruction filter bank and appropriate denoising algorithm can improve the performance of denoising. The experimental results revealed suitability of the newly developed wavelet to be the most appropriate wavelet basis function for denoising of fPCG signals in terms of MSE. The resultant denoised fPCG signals will preserve physiological information of diagnostic importance regarding health status of the unborn. 

## Figures and Tables

**Table 1:** Comparison of denoising algorithms.

Denoising algorithm	Code	MSE
Universal threshold algorithm with soft thresholding rule	sqtwolog(s)	0.8331
Universal threshold algorithm with hard thresholding rule	sqtwolog(h)	0.843
Minimax threshold algorithm with soft thresholding rule	minimaxi(s)	0.6295
Minimax threshold algorithm with hard thresholding rule	minimaxi(h)	0.7892
**Rigorous SURE threshold algorithm with soft thresholding rule**	**rigrsure(s)**	**0.575**
Rigorous SURE threshold algorithm with hard thresholding rule	rigrsure(h)	0.8165

**Table 2:** Comparison of different mother wavelets for denoising of fPCG signals in terms of MSE.

	Wavelet family										
				db5					coif4		
Algorithm↓	Signal→	S1	S2	S3	S4	S5	S1	S2	S3	S4	S5

(1) Sqtwolog(s)		0.8331	0.9124	1.099	0.3274	0.3328	0.5826	0.5744	1.1137	0.2615	0.2817
(2) Sqtwolog(h)		0.843	0.9124	1.099	0.3274	0.3328	0.5906	0.5666	1.1137	0.2615	0.2817
(3) Minimaxi(s)		0.6295	0.9073	1.099	0.3274	0.3328	0.5371	0.5716	1.1137	0.2712	0.2817
(4) Minimaxi(h)		0.7892	0.8953	1.099	0.7691	0.7779	0.8199	0.7972	1.1137	0.8162	0.8092
(5) Rigrsure(s)		0.575	0.8875	1.1001	0.4331	0.4396	0.4661	0.5531	1.081	0.2539	0.2669
(6) Rigrsure(h)		0.8165	0.8055	1.2945	0.7476	0.7561	1.0091	0.8633	1.2254	1.0062	0.8851

	Wavelet family										

				sym7					**Fetal (New)**		
Algorithm↓	Signal→	S1	S2	S3	S4	S5	S1	S2	S3	S4	S5

(1) Sqtwolog(s)		0.5608	0.6064	1.1245	0.2663	0.2769	0.5723	0.5766	1.1054	0.2712	0.2865
(2) Sqtwolog(h)		0.5629	0.5934	1.1245	0.2663	0.2769	0.6001	0.5613	1.1054	0.2712	0.2865
(3) Minimaxi(s)		0.5615	0.602	1.1245	0.2877	0.2837	0.5298	0.5743	1.1054	0.2712	0.2865
(4) Minimaxi(h)		0.836	0.8363	1.5005	0.8267	0.8224	0.8263	0.7889	1.1054	0.8133	0.8324
(5)** Rigrsure(s)**		0.5033	0.5979	1.1673	0.4122	0.4436	**0.4212**	**0.539**	**0.9998**	**0.2469**	**0.2518**
(6) Rigrsure(h)		0.8431	0.8394	1.5005	0.8369	0.8353	1.1776	0.8519	1.2173	1.1123	0.8732
